# Professional antigen presenting cells in human herpesvirus 8 infection

**DOI:** 10.3389/fimmu.2012.00427

**Published:** 2013-01-21

**Authors:** Emilee R. Knowlton, Lauren M. Lepone, Jun Li, Giovanna Rappocciolo, Frank J. Jenkins, Charles R. Rinaldo

**Affiliations:** ^1^Department of Infectious Diseases and Microbiology, Graduate School of Public Health, University of PittsburghPittsburgh, PA, USA; ^2^Department of Pathology, School of Medicine, University of PittsburghPittsburgh, PA, USA

**Keywords:** human herpesvirus 8, Kaposi's sarcoma, multicentric Castleman's disease, primary effusion lymphoma, dendritic cells, monocytes/macrophages, B lymphocytes, CD4 and CD8 T lymphocytes

## Abstract

Professional antigen presenting cells (APC), i.e., dendritic cells (DC), monocytes/macrophages, and B lymphocytes, are critically important in the recognition of an invading pathogen and presentation of antigens to the T cell-mediated arm of immunity. Human herpesvirus 8 (HHV-8) is one of the few human viruses that primarily targets these APC for infection, altering their cytokine profiles, manipulating their surface expression of MHC molecules, and altering their ability to activate HHV-8-specific T cells. This could be why T cell responses to HHV-8 antigens are not very robust. Of these APC, only B cells support complete, lytic HHV-8 infection. However, both complete and abortive virus replication cycles in APC could directly affect viral pathogenesis and progression to Kaposi's sarcoma (KS) and HHV-8-associated B cell cancers. In this review, we discuss the effects of HHV-8 infection on professional APC and their relationship to the development of KS and B cell lymphomas.

## Introduction

Human herpesvirus 8 (HHV-8) or Kaposi's sarcoma (KS)-associated herpesvirus, is the etiologic agent of KS (Chang et al., 1994), a neoplasm of endothelial origin that occurs in four distinct epidemiologic forms (Dedicoat and Newton, 2003; Jessop, 2006): classic or Mediterranean KS, epidemic or AIDS-related KS, endemic or African KS, and iatrogenic or organ transplant-associated KS. KS is the most common cancer associated with HIV-1 infection and AIDS (Cohen et al., 2005). Although the incidence of KS in HIV-1 infected persons declined with the advent of antiretroviral therapy (ART) (Gallafent et al., 2005), KS can occur in persons on ART with suppressed HIV-1 infection (Maurer et al., 2007). The success of ART in treating HIV-1-associated KS has also been countered by the occasional occurrence of an immune reconstitution inflammatory syndrome (Feller et al., 2008). This is a severe, temporary enhancement of KS lesions due to an increase in inflammation and immunologic recovery after ART.

The discovery of HHV-8 and its causal role in KS development opened the potential for prophylaxis and treatment of the infection and cancer with antiviral drugs, and prevention of both with a vaccine. Strategies to achieve these ends require an intimate knowledge of the pathogenesis and immune control of HHV-8 infection. We postulate that host control of HHV-8 infection and development of KS is linked to T cell interactions with HHV-8 infected, professional antigen presenting cells (APC), i.e., dendritic cells (DC), monocytes/macrophages, and B lymphocytes. Similarly, APC-T interactions are likely to be centrally involved in the HHV-8-associated B cell neoplasms multicentric Castleman's disease (MCD) (Soulier et al., 1995) and primary effusion lymphoma (PEL) (Cesarman et al., 1995; Nador et al., 1996). This review covers various aspects of HHV-8 targeting of APC and T cell responses in host control of this infectious and oncogenic process.

HHV-8 has been reported to be transmitted to common marmosets and cause persistent infection with rare, KS-like skin lesions (Chang et al., 2009). However, there is as yet no consensus that this or other simian models (Jung et al., 1999; Fickenscher and Fleckenstein, 2001; Rosenwirth et al., 2011) recapitulate human HHV-8 infection and development of KS or other cancers associated with this herpesvirus. Thus, although *in vitro* models are suspect to lacking certain *in vivo* characteristics, we will focus in this review on HHV-8 infection of human APC as being the most relevant to this human species-specific herpesvirus.

## HHV-8 infection of professional APC

As with the other human gamma herpesvirus, Epstein Barr virus (EBV) (Ning, 2011), HHV-8 targets APC both *in vivo* and *in vitro*. Indeed, the primary tropism of B cells by these gamma herpesviruses is uncommon among human virus infections. This sets the stage for development of their associated cancers both indirectly, through alteration of host immunity dependent on APC function, and directly via neoplastic effects of the virus. HHV-8 infection within KS tumors is primarily found in spindle cells, which are of mixed vascular and lymphatic endothelial cell and macrophage origin (Regezi et al., 1993; Bubman and Cesarman, 2003). However, HHV-8 is also detected in monocytes that are found in proximity to KS lesions, and circulating B cells of KS patients (Blasig et al., 1997; Monini et al., 1999). In PEL, HHV-8 is found in immunoblastic cells expressing plasma cell markers, and in plasmablastic cells of a less terminally differentiated state in MCD (Du et al., 2007). The intimate association of HHV-8 with such professional APC in the KS lesion and in other HHV-8-associated cancers suggests a major role for virus-APC interplay. Moreover, anti-HHV-8 T cell immunity that presumably is critically dependent on such virus-APC interactions, is present in HIV-1 infected and uninfected persons who are seropositive for HHV-8 (Robey et al., 2010). Achieving a better understanding of the role of HHV-8 in inducing-associated cancers could greatly benefit from a yet-to-be-developed *in vitro* model of primary HHV-8 infection of a natural target cell. This model should consistently reflect HHV-8 lytic, latent, and reactivation infections. HHV-8 infection of APC could provide such a model.

### HHV-8 receptors on APC

Infection of APC *in vitro* reveals different cycles of HHV-8 replication that are likely to relate to pathogenesis of the virus. HHV-8 initially targets cell surface receptors for infection, which represent the first level of APC alteration. Herpesviruses use more than one receptor to infect the same cell (Heldwein and Krummenacher, 2008). Use of these receptors by herpesviruses is hierarchical, based largely on differential expression of the receptors in specific cell types and states of cell activation. Extensive *in vitro* evidence indicates that the ubiquitous cell surface proteoglycan, heparan sulfate, serves as an initial binding receptor for HHV-8 on endothelial cells and fibroblasts, as well as APC (Akula et al., 2001b, 2002; Chandran, 2010; Kerur et al., 2010). Multiple integrins are subsequently involved in HHV-8 binding and entry (Kerur et al., 2010). A third level of differential selection has been identified from *in vitro* studies of the three major types of professional APC. The type II C-type lectin, DC-specific ICAM-3 grabbing nonintegrin (DC-SIGN; CD209) serves as a receptor for HHV-8 on both DC and B cells (Rappocciolo et al., 2006, 2008). Recently a new entry receptor for HHV-8 has been discovered on endothelial and epithelial cells (Hahn et al., 2012), i.e., ephrin receptor tyrosine kinase A2. This tyrosine kinase functions in neovascularization and oncogenesis, and has not yet been assessed in HHV-8 infection of APC.

The role of HHV-8 binding to APC receptors for entry and infection is being clarified with accumulating evidence that certain C-type lectins and integrins are essential to this process. For example, the Raji B lymphoblastoid cell line (LCL) and the myeloblastoid K562 erythroleukemia cell line constitutively express little or no DC-SIGN or α_3_β_1_ integrin (Rappocciolo et al., 2006). Thus, these cell lines do not support detectable production of HHV-8 virions (Blackbourn et al., 2000b; Bechtel et al., 2003; Rappocciolo et al., 2006). However, transfection of the cell lines with DC-SIGN renders them highly permissive for HHV-8 infection as measured by production of viral proteins and DNA (Rappocciolo et al., 2006). Moreover, infection of these cell lines can be blocked by anti-DC-SIGN mAb, soluble DC-SIGN, and mannan, a natural ligand of DC-SIGN. Interestingly, four B cell lines (BJAB, Ramos, BCBL1, JSC1) and two T cell lines (Jurkat and SupT1) are susceptible to infection through cell-mediated transmission with a doxycyline (DOX)-inducible cell line harboring recombinant HHV-8 (rKSHV.219) (Myoung and Ganem, 2011c). This indicates that viral entry can be achieved despite lack of expression of a major HHV-8 receptor. There is also evidence that HHV-8 can infect CD34^+^ stem cell precursors of DC *in vitro* by as yet undefined receptors (Henry et al., 1999; Larcher et al., 2005). It is likely that there are less prominent alternative receptors for HHV-8 that account for a small percentage of DC-SIGN negative APC and cell lines that can be infected by this virus.

### B cell infection with HHV-8

Suggestive evidence that HHV-8 is B-cell tropic *in vivo* is that HHV-8 DNA is detected in B cells from patients with KS lesions (Ambroziak et al., 1995) and some HIV-1/HHV-8 coinfected individuals (Murayama et al., 1994). Further evidence that HHV-8 targets B cells is the isolation of immortalized B cell lines from patients with PEL that are infected with HHV-8 (Cesarman et al., 1995). The first *in vitro* evidence that HHV-8 can infect B cells was that virus produced by these PEL cell lines could be transmitted to neonatal cord blood B cells (Mesri et al., 1996). We speculate that the lack of further evidence for B cell infection in those early years was that such infection requires DC-SIGN expression that is enhanced by an activated state in B cells. Thus, we showed that once blood-derived B cells are activated to express DC-SIGN, HHV-8 can effectively establish infection and elicit full-cycle production of infectious virions in these cells (Rappocciolo et al., 2008).

The fact that HHV-8 cannot infect Raji LCL or K562 cells expressing DC-SIGN that lacks its transmembrane domain supports that viral entry requires DC-SIGN-mediated endocytosis. Moreover, infection can be blocked by pretreatment of B cells with anti-DC-SIGN monoclonal antibody (mAb) or mannan, but not Ab specific for the amino acid transporter protein xCT (Rappocciolo et al., 2008). HHV-8 has been reported to use xCT for infection of surface adherent human cells (Kaleeba and Berger, 2006) and in a post-entry stage of human endothelial cell infection as part of a complex of heterodimeric membrane glycoprotein CD98 and the α3β1 and αVβ3 integrins (Veettil et al., 2008).

Notably, HHV-8 infection is not restricted to blood-derived B cells, as tonsillar B cells constitutively express DC-SIGN and can be lytically infected with the virus *in vitro* (Rappocciolo et al., 2008; Myoung and Ganem, 2011a). It is probable that B cells in such inflamed tonsillar tissue are in an endogenously activated state, resulting in enhanced expression of DC-SIGN.

The *in vitro* activated, B cell model for measuring HHV-8 infectivity and replication supports the concept that DC-SIGN is a major receptor for this virus (Rappocciolo et al., 2006, 2008; Chandran, 2010). This adds to the wealth of evidence that shows that, in contrast to previous reports (Ganem, 2007), DC-SIGN is required for highly efficient infection of natural APC targets with HHV-8. This is in addition to certain integrins that are also involved in HHV-8 entry (Akula et al., 2001a,b, 2002; Birkmann et al., 2001; Veettil et al., 2008). However, there is still need for improved reliable, quantitative measures of HHV-8 replication to better define B cell infection. These should include combinations of real time polymerase chain reaction (PCR) assays for cell-associated and non-cell-associated copy numbers of HHV-8 encapsidated DNA, flow cytometry assays for enumerating the number of monoclonal Ab (mAb)-stained cells expressing viral lytic and latency cycle proteins, and most important, cell culture-based assays for quantitating the number of infectious virus particles, e.g., a 50% tissue culture infectious dose assay. It is remarkable that almost 20 years after discovery of HHV-8 we do not have such basic assays to study the virus.

**Figure 1:**
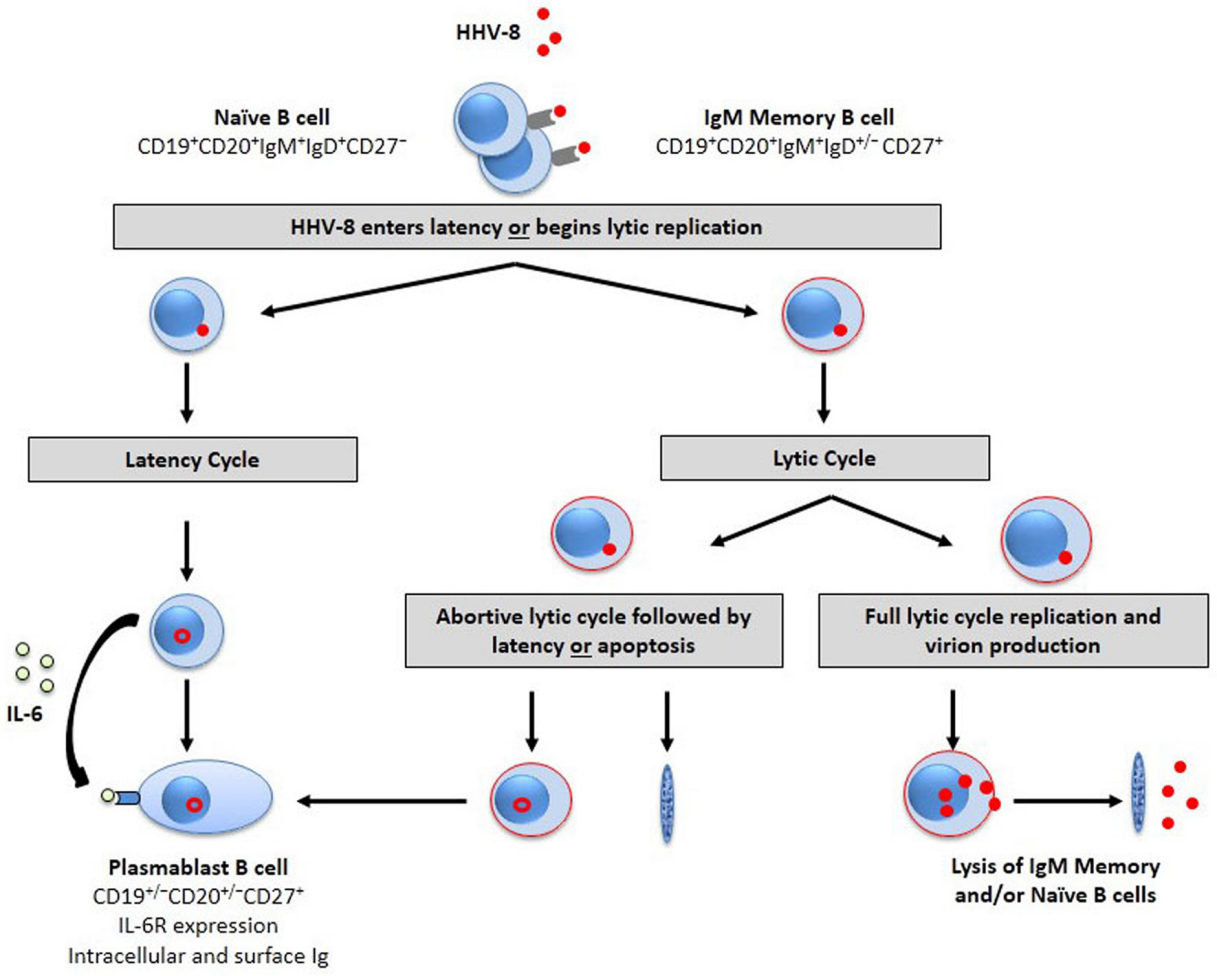
**HHV-8 targets B cell subpopulations for infection.** The B cell target for HHV-8 infection is unknown. However, evidence suggests that naïve and/or IgM memory B cell subsets are susceptible to HHV-8 infection. HHV-8 is endocytosed after binding to cell surface entry receptors. The virus then enters latency (left) or initiates lytic replication (Rappocciolo et al., 2008). Latently infected cells drive differentiation toward a plasmablast phenotype that is responsive to the proliferative cytokine, IL-6. The alternative pathway is the entry of HHV-8 into the lytic cycle to begin transcription of lytic-associated proteins in activated B cells (red outline). The lytic cycle may stop prior to virion production, resulting in an abortive replicative cycle as seen in DC, endothelial cells, and fibroblasts. The virus in these cells likely enters latency or may result in B cell apoptosis. Some cells, however, will support full lytic cycle replication, resulting in lytic protein synthesis, and increases in viral DNA that correspond to infectious HHV-8 progeny and subsequent release through cell lysis.

It is postulated that HHV-8 infection drives B cells to an early plasmablast-like state in MCD and a preterminal plasma cell stage of differentiation in PEL (Frizzera et al., 1983; Miller et al., 1984; Cesarman et al., 1995; Nador et al., 1995; Agematsu et al., 1997; Matolcsy et al., 1998; Dupin et al., 2000; Du et al., 2001; Klein et al., 2003; Chadburn et al., 2004, 2008; Hassman et al., 2011). Hassman et al. (2011) recently showed that latency-associated nuclear antigen (LANA)^+^ B cells express IgM and the λ light chain at 2.5–3.5 days post-HHV-8 infection *in vitro*. These cells are plasmablast-like with increased interleukin (IL) 6 receptor expression and increased proliferative response to IL-6, with 7–36% expressing CD27. This molecule is a member of the tumor necrosis factor (TNF)-receptor superfamily, and is involved in regulation of B cell activation. It is not known whether HHV-8 directly infects these IgM^+^ memory B cells or a precursor of these cells. Also, there are no data on which subset of B cells supports a complete lytic cycle of replication with virion formation and death of the cell. Likewise, there is the possibility that HHV-8 infection in these B cell subsets results in an abortive replication cycle, leaving memory B cells that survive and maintain latent virus infection. We speculate that HHV-8 infection of naïve and IgM memory B cells leads to establishment of latency in a portion of cells, resulting in virus-driven plasmablast differentiation, while some cells support the viral lytic cycle (Figure 1).

Currently, the main *in vitro* models that recapitulate HHV-8 infection of APC are B lineage cell lines persistently infected with the virus. Among them, the body cavity-based lymphoma cell line (BCBL-1), a B cell line derived from a patient with PEL which is latently infected with HHV-8 and EBV negative (Chang et al., 1994; Cesarman et al., 1995), is the most commonly utilized. In latently infected cells of KS tumors and in such cell line models, the virus exists as circular or episomal DNA with limited expression of latent genes (Schulz, 2006). Therefore, HHV-8 lytic and latent infections cannot be defined conventionally in these systems starting with the total absence of infectious viral particles, as there is always a low level of persistent virus production. However, latency can be disrupted, triggering the lytic cascade of viral replication, and lytic genes expressed sequentially as immediate early (IE) genes, early (E) genes, and late (L) genes, resulting in production of encapsidated virions. Such lytic viral replication is largely irreversible (Chandran, 2010; Wen and Damania, 2010).

HHV-8 lytic gene profiling in these persistently infected PEL cell lines has been extensively accomplished using tiling microarray (Chen et al., 2009), DNA microarray (Agematsu et al., 1997; Chandriani et al., 2010; Lock et al., 2010), and high-throughput real-time PCR (Fakhari and Dittmer, 2002; Dittmer, 2003). However, most studies on latency-lytic reactivation of HHV-8 use various chemicals to induce viral replication (Myoung and Ganem, 2011b). The question is whether such reactivation reflects natural HHV-8 viral lytic reactivation from latency, since chemical agents such as 12-*O*-tetradecanoylphorbol-13-acetate (TPA) have pleiotropic effects on host cell signaling and chromatin structure. How they affect cell signaling pathways is also unknown. Thus, the natural reactivated cascade of lytic transcripts of HHV-8 still waits to be revealed.

Instead of using chemical inducers, Nakamura et al. (2003) developed an engineered BCBL-1 cell line that inducibly expresses the replication transactivator protein, RTA, encoded by ORF50, i.e., TREx-BCBL1-RTA. RTA is necessary and sufficient for the switch between HHV-8 latency and lytic replication (Deng et al., 2000). In fact, mutation of the RBP-Jk sites within the RTA promoter is enough to enhance latency in transformed-293 cells and peripheral blood mononuclear cells (PBMC) (Lu et al., 2012). In the TREx-BCBL1-RTA cell line, RTA expression is under the control of a DOX-inducible promoter. Treatment of TREx-BCBL1-RTA cells with DOX results in expression of RTA, which in turn induces viral replication (Nakamura et al., 2003). While the role of RTA in causing a switch from latency to viral replication has been demonstrated, the mechanisms regulating coordinate induction of expression of most of the HHV-8 lytic genes during this reactivation have not been evaluated in a systematic fashion.

Virus reactivation events have been studied in primary and immortalized microvascular endothelial cells (MVEC) with the recombinant virus, rKSHV.219 (Vieira and O'Hearn, 2004). This virus expresses a green fluorescent protein (GFP) under an EF-1α promoter to indicate infection and a red fluorescent protein under a PAN promoter to indicate lytic transcription. This model should be considered for a more detailed evaluation of APC infection and reactivation.

Transcription of HHV-8 lytic genes occurs during either a primary infection of susceptible cells or during reactivation of latently infected cells. The question remains whether the kinetic gene activation in a chemically induced cell line (BCBL-1) or the naturally targeted RTA (TREx-BCBL1-RTA) will reflect the cascade events of natural infection of B cells or other APC. To identify the true gene transcription and reactivation events in HHV-8 infection, primary cells susceptible to HHV-8 should be used. Only then can the observations from TPA-induced BCBL-1 and DOX-induced TREx-BCBL1-RTA cell lines be validated.

In 2005, a cluster of microRNA (miRNA) coded by HHV-8 was discovered (Cai et al., 2005; Samols et al., 2005). This short, 22 nucleotide, non-coding miRNA silences mRNA expression through a silencing complex (miRISC). HHV-8 miRNAs are expressed during lytic and latency cycles of virus replication, and act on both cellular and viral transcriptomes (Gottwein, 2012). Studies of HHV-8 miRNA have utilized PEL cell lines, as well as foreskin fibroblasts and endothelial cells. These indicate a multifactorial role in maintaining viral latency, regulating lytic virus replication, and enhancing cell survival. As miRNA activity is dependent on its level and targets within specific cell types, it is imperative that miRNA be assessed in primary B cells.

Finally, as HHV-8 is one of the few human viruses that primarily targets B cells, an in depth understanding of the effects HHV-8 infection has on these cells should be established. However, little data exist concerning B cell activation states or surface marker expression upon HHV-8 infection. Likewise, interactions between HHV-8 infected B cells and CD4^+^ T helper cells are yet to be defined. Considering that the major function of B cells is production of Ab that prevent and ameliorate infection, there is need to assess the quality and quantity of Ab production over the course of HHV-8 infection and development of KS. Yet, there is no consensus assay for detecting or titering anti-HHV-8 Ab. Detection of anti-LANA Ab by immunofluorescence assays has low sensitivity (as low as 64%) among individuals with KS (Rabkin et al., 1998; Corchero et al., 2001; Nascimento et al., 2007). ELISA and Western blot assays for anti-latent (LANA) and lytic (ORF65 or K8.1) Ab have higher sensitivities and are often used for serologic testing, yet can have low specificities (Nascimento et al., 2007). These conventional methods for serologic testing therefore lack standardization and can be unreliable, underlining the necessity for more accurate methods of quantifying anti-HHV-8 Ab.

Regardless of the vagaries of anti-HHV-8 Ab assessments, humoral immunity to HHV-8 infection has been described for several cohorts. A luciferase immunoprecipitation system that quantifies Ab response to multiple antigens was used to compare profiles of KS, MCD, and PEL patients (Burbelo et al., 2010). The study showed significant differences in Ab responses among the groups, including higher anti-K8.1 Ab detected in PEL and MCD compared to KS and higher Ab titers to ORF65 in PEL compared to KS. Likewise, higher Ab titers against v-cyclin were observed in KS and PEL compared to MCD, and higher anti-LANA Ab titers were detected in KS compared to MCD. An explanation for the difference in Ab responses in individuals with these HHV-8-associated cancers is currently unknown, but is likely a reflection of the differential expression of latent and lytic viral genes. The quality and quantity of anti-HHV-8 response may change over the course of disease progression or after anti-viral therapy. Following ART, increases in Ab against both latent and lytic proteins have been observed for individuals with or without KS (Gill et al., 2002; Wilkinson et al., 2002; Bourboulia et al., 2004; Sullivan et al., 2010). More in depth studies with larger cohorts and advanced testing methods should be performed, while *in vitro* models for HHV-8 infection in B cells and detection of antiviral Ab should be established.

Interestingly, there are only minimal data on neutralizing Ab in HHV-8 infection. The first such evidence was that rabbit polyclonal neutralizing Ab to gB prevent HHV-8 infection of primary human foreskin fibroblasts (Akula et al., 2001a) and oral epithelial cells (Duus et al., 2004). Concurrently, it was demonstrated that sera from persons who were seropositive for HHV-8 as shown by anti-LANA immunofluoresence assay also had neutralizing Ab that inhibited virus infection of transformed dermal MVEC (Dialyna et al., 2004). Using a recombinant HHV-8 (rKSHV.152) that expresses GFP, Kimball et al. (2004) found significantly lower neutralizing Ab titers to HHV-8 in the serum of HIV-1 infected persons with KS compared to those without KS. This is in contrast to Inoue et al. (2004) who reported that there were no differences in neutralizing Ab titers between HIV-1 infected patients with or without KS. However, the latter study used an HHV-8 reporter cell line T1H6 treated with polybrene in their virus neutralization assay. Polybrene results in receptor-independent infection (Davis et al., 2002), thus potentially obscuring interpretation of virus neutralization assays. Finally, it is evident that there is a need for in depth, longitudinal studies of neutralizing Ab and other antiviral Ab such as those that mediate Ab-dependent cell cytotoxicity, in relation to progression of HHV-8 infection and development HHV-8 related cancers.

### Monocyte/macrophage infection with HHV-8

Macrophages in several body compartments naturally express DC-SIGN (Granelli-Piperno et al., 2005; Kamada et al., 2009), as well as integrins including α_3_β_1_ (Ammon et al., 2000), which presumably renders them susceptible to HHV-8 infection. An early report showed that monocyte-derived macrophages (MDM) from normal donors that are stimulated *in vitro* with allogeneic PBMC can be infected by HHV-8, but this rarely resulted in complete, lytic replication (Blackbourn et al., 2000b). In addition, treatment of blood monocytes from KS patients with proinflammatory cytokines *in vitro* results in HHV-8 persistence (Monini et al., 1999).

MDM become susceptible to HHV-8 infection *in vitro* after activation with IL-13, which enhances DC-SIGN expression (Rappocciolo et al., 2006). IL-13 is an anti-inflammatory, Th2 cytokine that induces alternatively activated (M2) macrophages (Hao et al., 2012). These contrast with classically activated (M1) macrophages which have preferential expression of proinflammatory cytokines, chemokines, and effector molecules, such as IL-12, IL-23, tumor necrosis factor α (TNF-α), inducible nitric oxide synthase (iNOS), and MHC class I and II. In contrast, M2 macrophages express a wide array of anti-inflammatory molecules, including IL-10, and transforming growth factor β (TGF-β). IL-13 also promotes differentiation of B cells into Ab-secreting plasma cells. M2 macrophages express more DC-SIGN than M1 macrophages (Cassol et al., 2012), supporting the concept that M2 macrophages are likely to serve as a more efficient target cell for HHV-8 infection. Importantly, multiple receptors are needed for efficient infection of these macrophages by HHV-8. Indeed, when DC-SIGN is blocked in IL-13-activated MDM or the monocytic cell line THP-1, HHV-8 can still bind using heparan sulfate, although virus entry is reduced (Kerur et al., 2010).

Although studies are lacking for coordinated expression of HHV-8 ORFs in monocytes/macrophages, HHV-8 establishes productive infection in THP-1 cells with an ordered expression of latency gene ORF73 and lytic gene ORF50. In fact, the HHV-8 genome was reported to persist for 30 days in these cells (Kerur et al., 2010). Such limited expression of lytic genes together with the persistence of latency genes is believed to be unique for HHV-8 (Krishnan et al., 2004).

Of interest is that ORF K14 of HHV-8 encodes a surface glycoprotein vOX2 that is homologous to cellular OX2 (Chung et al., 2002), and which inhibits macrophage function (Foster-Cuevas et al., 2004). The vOX2 glycoprotein could be central to HHV-8 immunopathogenesis in that it stimulates production of inflammatory cytokines IL-1β, IL-6, monocyte chemoattractant protein 1 (MCP-1), and TNF-α in primary monocytes, MDM, and monocyte-derived DC (MDDC) (Chung et al., 2002). Furthermore, expression of vOX2 on B cells stimulates monocytes to produce inflammatory cytokines. MDM transfected with vOX2 produce inflammatory cytokines and have enhanced phagocytic activity, while inhibiting the immunomodulatory effects of IFN-γ and down-regulating MHC class I and class II expression on macrophages (Salata et al., 2009). It was recently reported that vOX2-transfected APC co-cultured with T cells results in suppressed IFN-γ production and mobilization of the cytolytic granule marker CD107a through inhibition of ERK1/2 phosphorylation (Misstear et al., 2012).

### HHV-8 infection of myeloid DC

Evidence of infection of human DC *in vivo* with HHV-8 has been limited (Rettig et al., 1997; Olsen et al., 1998). However, there is no *a priori* reason why human DC should not take up HHV-8 and support at least abortive infection *in vivo*. Tissue resident, myeloid DC constitutively express DC-SIGN (Soilleux et al., 2002). MDDC express DC-SIGN *in vitro*, again indicating a central role for this receptor in HHV-8 infection of APC. Studies are needed to determine whether this C-type lectin is required for HHV-8 infection of tissue DC *in vivo*.

When MDDC are infected *in vitro* with HHV-8, viral lytic proteins are produced with little viral DNA production (Rappocciolo et al., 2006), similar to abortive HHV-8 infection of vascular endothelial cells (Renne et al., 1998; Vieira et al., 2001; Akula et al., 2002; Naranatt et al., 2003; Raghu et al., 2009). Although HHV-8 infection does not significantly alter MDDC viability, it decreases MDDC function, i.e., lowers their capacity to activate antigen-specific CD8^+^ T cell responses. Moreover, HHV-8 infected MDDC have impaired antigen uptake, with a significant decrease in endocytic capacity and DC-SIGN expression within 24 h after infection. DC-SIGN internalization in MDDC is associated with lytic HHV-8 gene expression (Rappocciolo et al., 2006). In addition to MDDC, HHV-8 *in vitro* infection of IL-13-treated MDM results in a loss of DC-SIGN surface expression, suggesting that HHV-8 binding to DC-SIGN triggers internalization. Hence, alteration of DC-SIGN expression could be a strategy used by HHV-8 to escape immune defenses and lead to a non-robust immune response (Wang et al., 2002).

### HHV-8 infection of langerhans cells (LC) and interstitial-dermal DC (iDDC)

The skin and mucosa contains two major types of DC: (1) langerhans cells (LC), which reside in the epidermis in close contact with keratinocytes and (2) interstitial-dermal DC (iDDC), resident in the dermis and mucosal layers. LC and iDDC process cutaneous antigens and migrate to draining lymph nodes to present antigens to T and B cells. Because of the strategic position of LC and iDDC and their ability to capture pathogens, these cells could represent potential targets for HHV-8 infection. Furthermore, due to the expression of the C-type lectins, i.e., langerin (CD207) and DC-SIGN, on LC and iDDC, respectively, it is tempting to speculate that HHV-8 could utilize the same entry mechanisms as seen in MDDC (Rappocciolo et al., 2006). Therefore, it is important to determine if these APC are also targeted by HHV-8 and whether they support full lytic replication or an abortive cycle. LC and iDDC can be generated from pluripotent cord blood CD34^+^ cells (Caux et al., 1997), which could prove to be valuable tools to study HHV-8 infection and subsequent antigen process and presentation to T cells (Colleton et al., 2009).

### HHV-8 infection of plasmacytoid DC (pDC)

Plasmacytoid DC (pDC) are a lymphoid-lineage subset of APC that produce extraordinary amounts of the antiviral protein interferon α (IFN-α) in response to virus infection (Liu, 2005). Although pDC do not express DC-SIGN, they are susceptible to *in vitro* infection with GFP-tagged HHV-8 (West et al., 2011). GFP expression was detected in 23% of pDC from healthy blood donors at 16 h post-infection. Also, ORF57 and LANA expression was detected by PCR at 48 and 72 h, respectively. Infection of the pDC results in upregulation of activation molecule CD83 and T cell co-receptor CD86, and induces production of IFN-α. Induction of IFN-α by HHV-8 occurs through activation of Toll-like receptor 9 (TLR9) signaling in pDC. At present, however, it is unclear what receptors HHV-8 uses to infect pDC, and whether HHV-8 infection of pDC results in an abortive or fully lytic viral replicative cycle.

### HHV-8 infection and TLR

Several types of TLR expressed on different APC are emerging as important factors in the innate and adaptive immune response to HHV-8. Notably, virus triggering of C-type lectins, including DC-SIGN, in combination with TLR triggering on DC induces signaling and cytokine responses. These in turn regulate T cell polarization that is central to host immune control of infections (Van Kooyk, 2008). In addition, TLR have also been implicated in reactivation of HHV-8. TRL7/8 could control reactivation of HHV-8 from latency in B cells. That is, agonists specific for TLR7/8 reactivate latent HHV-8 and induce viral lytic gene transcription and replication in latently infected PEL cell lines of B cell origin (Gregory et al., 2009). This has important implications for host control of HHV-8 infection, as signaling through the TLR1/2/6 complex, TLR7, TLR9, and TLR10 affects multiple stages of B cell activation, proliferation, cytokine secretion, terminal differentiation, and Ab secretion in response to T cell-dependent antigens (Bekeredjian-Ding and Jego, 2009).

## Summary

Utilizing heparan sulfate, cell surface integrins, and DC-SIGN for binding and entry, HHV-8 establishes infection in professional APC that are essential to processing and presenting antigen. As these HHV-8-targeted APC initiate T and B cell adaptive immune responses, this could demonstrate an evolutionary mechanism to establish viral latency and persistence in the host. Accordingly, regulation of viral protein expression limits detection of HHV-8 in APC by T cells, resulting in sustained latency. Although valuable conclusions have been drawn from immortalized cell lines as surrogates for these APC, primary cell models such as blood and tissue APC provide a more natural account of the quality of HHV-8 infection, better displaying the mechanisms of latency and abortive and non-abortive virus replicative cycles. This is reflected by the fact that among professional APC, HHV-8 undergoes full lytic replication only in activated B lymphocytes, yet can bind to, enter and alter various functions of DC and monocytes/macrophages.

## Cytokines and chemokines in HHV-8 infection of APC

Cytokines and chemokines produced by inflammatory APC, as well as T cells, play a crucial role in HHV-8 replication and development of KS. Inflammatory changes occur early in KS prior to the detection of the cancer (Mesri et al., 2010). Proinflammatory processes drive early stage KS to develop into mature, spindle cell lesions (Rezaee et al., 2006). Thus, KS tumors are comprised of spindle-shaped cells of endothelial origin (Regezi et al., 1993) in an environment rich in inflammatory cell infiltrates, including B cells, monocytes/macrophages, and CD8^+^ T cells (Monini et al., 1999). The infiltrating cells produce large amounts of Th1 polarizing, proinflammatory cytokines (e.g., IFN-γ, IL-1β, TNF-α and IL-6), chemokines (e.g., IL-8), and growth factors [e.g., vascular endothelial growth factor (VEGF)], which can induce the KS-like phenotype observed in activated endothelial cells (Fiorelli et al., 1998; Monini et al., 1999; Ensoli et al., 2000). IFN-γ is the earliest and most abundant inflammatory cytokine observed in KS (Fiorelli et al., 1998) and can be detected in KS lesions before evidence of HHV-8 DNA (Monini et al., 1999). IL-6 is also found at very high levels in both KS lesions and in circulation of patients with MCD (Ambroziak et al., 1995). In MCD, IL-6 induces B cell proliferation and causes inflammatory clinical symptoms (Schulte and Talat, 2010). Observations from a transgenic mouse model demonstrate that mice expressing viral IL-6 but lacking mammalian IL-6 do not experience phenotypic changes (e.g., lymphoadenopathy, hypergammaglobulinemia, splenomegaly) associated with MCD (Suthaus et al., 2012). IL-6, as well as oncostatin M (OSM) and IL-10, are also detected at high levels in PEL cells. Proliferation of PEL can be inhibited when receptors for the IL-6 pathway are blocked (Drexler et al., 1999). Thus, an as yet minimally detailed imbalance in the Th1-Th2 milieu during HHV-8 infection appears to be closely linked to APC in driving the outgrowth of KS endothelial cells, as well as PEL and MCD B cells.

Other cytokines and chemokines produced by APC, particularly IL-8 and MCP-1, are elevated in serum of KS patients and have been implicated in many cancers (Sun et al., 2006; Mehrad et al., 2007). Enhanced expression of MCP-1, but not other NF-kβ activated cytokines (RANTES, IL-8 and TNF-α), is also detected in *in vitro* infected human umbilical vein endothelial cells (HUVEC) (Caselli et al., 2007). When bound to its CCR2 receptor on endothelial cells, MCP-1 results in chemotaxis and mediates angiogenesis *in vitro* (Galvez et al., 2005; Mehrad et al., 2007). KS tumors are highly vascularized with abnormal angiogenesis, leading to enhanced blood flow to the tumor by expanding pre-existing blood vessels (Mesri et al., 2010). IL-1β, TNF-α, IL-8, and IL-6 can also enhance tumor cell growth and vascularization (Ensoli et al., 1989; Ensoli and Sturzl, 1998; Fiorelli et al., 1998) by inducing the expression of two angiogenic mediators, i.e., VEGF and fibroblastic growth factor (Ensoli et al., 1989; Cohen et al., 1996; Cornali et al., 1996; Monini et al., 1999). In addition to angiogenesis, inflammatory cells and cytokines can contribute to viral reactivation and replication. IFN-γ was shown to induce ORF59 expression in BCBL-1 (Blackbourn et al., 2000a) and reactivate latent HHV-8 in BC-3 PEL cells by activation of Pim-family kinases (Cheng et al., 2009). Mercader et al. showed OSM, IFN-γ, and hepatocyte growth factor/scatter factor-induced lytic cycle activation of BCBL-1 resulting in virion production (Mercader et al., 2000). This principle has been demonstrated in HHV-8 infected PBMC, where inflammatory cytokines could maintain or increase viral load up to 10-fold when the infected cells were cultured in the presence of inflammatory cytokines (Monini et al., 1999).

In AIDS-related KS, immune dysregulation and induction of inflammatory cytokines acts to further enhance KS tumor growth. When BCBL-1 cells that are latently infected with HHV-8 are cultured with HIV-1 infected CD4^+^ T cells, soluble factors secreted by the T cells cause the virus to enter lytic reactivation (Mercader et al., 2000). Inflammatory cytokines induced by both HIV-1-infected and HHV-8-infected cells promote expression of receptors for HIV-1 Tat, which acts as a progression factor in KS development (Ensoli et al., 1990; Barillari et al., 1993) and increasing viral load (Harrington et al., 1997). Indeed, serum and cell samples taken from KS lesions of HIV-1 infected individuals co-infected with HHV-8 show markedly increased levels of inflammatory cytokines, growth factors, and angiogenic mediators (Ensoli and Sturzl, 1998; Pugliese et al., 2002).

HHV-8 has a broad cellular tropism *in vivo* including B cells, endothelial cells, monocytes, keratinocytes, and epithelial cells that could result in production of inflammatory mediators (Chakraborty et al., 2012). In pDC, HHV-8 induces enhanced levels of IL-6, IL-8, MIP-1α, MIP-1β, CCL22, and IFN-α (West et al., 2011). In monocytes, production of IP-10, IFN-β1, MCP-1, and IRF-1 occurs in conjunction with an upregulation of TLR3 expression (West and Damania, 2008). Our lab has previously demonstrated that MDDC infected *in vitro* with HHV-8 secrete IL-6, TNF-α, IP-10, MIP-1α, and MIP-1β (Hensler et al., 2009). While IL-12p40 expression increases post-infection, bioactive IL-12p70 is not detected in HHV-8 infected MDDC. This suggests a virus-related inhibition of constitutive production of IL-12p35, or a defect in complexing of these subunits into IL-12p70. Furthermore, the results support an intentional skewing of cytokine production in HHV-8-infected MDDC toward induction of a Th2 response that could enhance development of KS.

We speculate that cytokine and chemokine profiles in HHV-8 infected B cells is similar to the cytokine dysregulation observed in EBV-associated disease (Gosselin et al., 1992; Klein et al., 1996; Kurzrock, 2001; Glaser et al., 2006). Elevated levels of IL-1β, TNF-α, IL-6, IL-8, and IL-10 are detected in the serum of patients with EBV-associated diseases, while a less favorable outcome correlates with increases of IL-6 and IL-10 in Hodgkin's lymphoma (Fayad et al., 2001). Common strategies between EBV and HHV-8, such as NF-κ B signaling pathway alterations (Hayden and Ghosh, 2008; De Oliveira et al., 2010) and the expression of virokines (Sin and Dittmer, 2012), imply that an imbalance of immune mediators is associated with the oncogenesis of these gammaherpesviruses.

### HHV-8 encoded proteins involved in immune mediator responses

Besides cellular cytokines and chemokines, HHV-8 encodes several proteins that share homology to host genes. These are involved in inflammation and angiogenesis that contribute to the inflammatory environment observed in KS. Cytokines and chemokines encoded by HHV-8 have been the focus of numerous studies and reviews (Nicholas, 2005; Gasperini et al., 2008; Mesri et al., 2010; Sakakibara and Tosato, 2011; Lee et al., 2012; Sin and Dittmer, 2012). Thus, vIL-6 has 24% homology to human IL-6 and can induce expression of VEGF and MCP-1 (Nicholas et al., 1997). These in turn trigger angiogenic pathways. Elevated levels of vIL-6, as well as levels of human IL-6 and HHV-8 viral load, have been associated with a recently described syndrome of severe systemic inflammatory symptoms (Uldrick et al., 2010). However, consistently reproducible assays for quantitation of vIL6 are needed to extend such studies.

The G-protein coupled receptor (vGPCR) is an early lytic phase gene homologous to the IL-8 receptor, CXCR-2 (Arvanitakis et al., 1997; Nicholas, 2005). vGPCR constitutively signals and results in enhanced production of IL-1β, IL-8, MCP-1, IL-6, and VEGF that can have both autocrine and paracrine effects (Gershengorn et al., 1998; Schwarz and Murphy, 2001). K1 and K15 are signal transducing proteins that induce VEGF, IL-6, and IL-8 (Choi and Nicholas, 2010). LANA and the viral flice inhibitory proteins (vFLIP) have been linked to enhanced cytokine production via activation of the MAPK and NF-κ B pathways, respectively (Wang and Boshoff, 2005). vIRF3 expression inhibits MHC class II expression as well as IFN-γ production (Schmidt et al., 2011). Finally, viral macrophage inflammatory proteins (MIPs) (vCCL1, vCCL2, vCCL3) share homology to MIP1-α and RANTES and can induce monocyte chemotaxis and signal transduction (Arvanitakis et al., 1997; Nakano et al., 2003; Nicholas, 2005). Given the plethora of such data derived from highly manipulated molecular and cell line models, the challenge is to link these unique HHV-8 factors directly to HHV-8 infection and development of cancers in natural cellular targets of the virus.

## Summary

A delicate balance exists between protective immunity involving cytokine and chemokine production by host APC and virus-driven induction of cytokines and chemokines that aid in the dissemination of infection and mediate pathogenesis. Several of the mediators that are essential to the immune response and activation of lymphocytes can exacerbate infection and cause clinical symptoms when over produced in response to HHV-8 infection, including IFN-γ, IL-1β, IL-6, IL-8, TNF-α, and MCP-1. The role of each cytokine/chemokine in the development of KS and the inflammatory environment observed within KS tumors likely varies depending on their quantity and origin. Therefore, production of these immune mediators by cells the virus naturally targets for infection may better reflect HHV-8-induced, cytokine/chemokine driven pathogenesis.

## APC-T cell interactions in HHV-8 infection

Given our rudimentary understanding of HHV-8-APC interactions, we know even less regarding HHV-8-specific T cell-APC interactions and their role in controlling viral infection and disease. A key challenge is to adapt current *in vitro* models using cell lines and HHV-8 constructs to systems that allow deciphering of the basic steps of natural HHV-8 infection, and antigen processing and presentation, in various types of APC. The interactions of APC with T cells that underlie the generation of anti-HHV-8 T cell immunity begin with DC of myeloid origin that take up viral antigen at local sites of infection, then travel to the draining lymphatics, and induce antiviral T cell responses (Ueno et al., 2007). There are specialized subsets of DC that populate different tissue sites and have distinct virologic interactions and immunologic functions. Myeloid-derived LC and iDDC populate the epidermis and dermis respectively, and are associated with KS lesions. iDDC are similar in phenotype and function to dermal DC, and are linked to systemic KS lesions. Other DC subsets such as CD141^+^ DC which are the human surrogates of mouse CD8α DC subsets (Bachem et al., 2010; Jongbloed et al., 2010), could be natural targets for HHV-8. These cells exhibit strong priming of T cells to antigen. It is imperative that we assess transcription of HHV-8 ORFs in natural targets of the virus, in comparison to well-documented immunomodulatory properties of HHV-8 expressed in cell lines and artificial constructs, such as persistently infected BCBL-1 (Coscoy, 2007).

Interactions of HHV-8 with DC subsets could be critical at the site of virus replication, and be centrally involved in generating T cell responses to the virus. Efficient activation of HHV-8 epitope-specific CD8^+^ T cells requires presentation by peptide-loaded, autologous, mature DC (Wang et al., 2002). This is similar to optimal activation of anti-EBV CTL by peptide-loaded DC (Wheatley et al., 1998; Redchenko and Rickinson, 1999; Subklewe et al., 1999a,b, 2001, 2005; Lin et al., 2002). Other studies have revealed polyfunctional CD8^+^ and CD4^+^ T cell reactivity and new MHC class I epitopes for HIV-1 Gag and Nef using peptide-loaded DC (Huang et al., 2010). Importantly, we have used this DC model to map epitopes of HHV-8 lytic and latency proteins with libraries of synthetic, 15mer peptides overlapping by 11aa (Lepone et al., 2010). Nevertheless, it may be more practical to generate large numbers of CD40L-activated, autologous B cells that favorably compare to DC as APC (Schultze et al., 2004).

To date, relatively few CD8^+^ and CD4^+^ T cell epitopes within only 15 of the over 80 ORFs of HHV-8 have been identified, and most of these are restricted by HLA A^*^0201 (Robey et al., 2010). Information is therefore needed on the broad range of potential antigenic sites in the virus that are restricted by other MHC class I and II haplotypes. Moreover, no studies have yet established a hierarchy of naïve and memory CD8^+^ or CD4^+^ T cell responses to HHV-8 epitopes in control of HHV-8 infection. There also are minimal data on whether alterations in anti-HHV-8 T cell responses are related to development of KS (Guihot et al., 2006) and whether the lower incidence of KS in HIV-1 infected persons receiving ART is related to increases in anti-HHV-8 T cell responses (Bourboulia et al., 2004; Bihl et al., 2007a). Such information is important for development of prophylactic and therapeutic vaccines for HHV-8.

HHV-8 infection alters the capacity of DC to be recognized by and activate CTL. Both direct presentation using viral proteins endogenously produced in DC, and cross-presentation pathways using viral proteins from exogenous sources of virus are likely to be operative in HHV-8 infection. In fact, EBV does not replicate in MDDC, which instead activate anti-EBV CD8^+^ T cells by an antigen cross-presentation pathway (Herr et al., 2000; Subklewe et al., 2001; Popescu et al., 2003).

It is possible that HHV-8 infected, apoptotic endothelial cells, macrophages, and B cells are recognized as “distressed” cells at local sites of infection and engulfed by LC and iDDC (Ueno et al., 2007). These DC could then migrate to local lymph nodes while processing the ingested viral proteins through alternative MHC class I pathways for presentation to CD8^+^ T cells. Furthermore, several HHV-8 proteins, particularly those coded by ORF K3 and K5, have intriguing properties of altering expression of MHC class I, T cell coreceptors, and DC-SIGN. Interestingly, cytokines released by PELs can interfere with the *in vitro* differentiation of immature MDDC from CD14^+^ monocytes (Cirone et al., 2008).

An intriguing recent discovery is that activated CD4^+^ T cells suppress HHV-8 lytic replication in tonsillar B cells (Myoung and Ganem, 2011a). The suppressive activity requires cell-cell contact. However, it is not a classic CTL response, as it can be mediated by T cells from HHV-8 seronegative persons, is not MHC restricted and does not lyse the B cell targets. This is proposed to be a pathway by which HHV-8 is driven into latency in B cells. These CD4^+^ T cells are reminiscent of CD8^+^ T cells that exhibit non-cytotoxic responses that suppress HIV-1 infection (Killian et al., 2011).

### Altered HHV-8 antigen processing and presentation

Presentation of HHV-8 proteins to both CD8 (MHC class I restricted) and CD4 (MHC class II restricted) T cells is impaired by HHV-8 infection. Evidence suggests that anti-HHV-8 CD8^+^ T cell responses can be inhibited by K3 and K5 proteins that down-regulate MHC class I expression (Coscoy and Ganem, 2000; Ishido et al., 2000b). Interestingly, K5-encoded MIR2 down-regulates T cell costimulatory molecules ICAM-1 and CD86 (Coscoy and Ganem, 2001) and IFNγR1 (Li et al., 2007b) which could act to decrease T cell responses to HHV-8. Ishido et al. showed that K5 also dampens natural killer (NK) cell-mediated cytotoxicity by downregulation of ICAM-1 and CD86 (Ishido et al., 2000a). The NK activating receptor, NKG2D, responsible for detecting infected cells, is downregulated by HHV-8 K5 (Thomas et al., 2008) via the release of the tumor-associated prostaglandin E2 (PGE2) from KS cells (Dupuy et al., 2012). This also results in inhibition of IL-15-mediated NK cell activation and survival, adding to the immune escape tactics employed by this virus (Dupuy et al., 2012). Likewise, infection of primary fibroblasts results in limited NK cell activation and killing activity (Matthews et al., 2011). Brander et al. (2000), reported a decrease in lysis by HIV-1 peptide-specific CTL clones of cells infected with HHV-8. Thus, it is apparent that K3 and K5 have multifactorial effects on immune control of HHV-8 infection. Of note is that the intracellular load of HHV-8 in infected endothelial cells is directly related to their loss of expression of MHC class I and ICAM-1, in association with expression of MIR2 (Adang et al., 2007). Interestingly, EBV infection also decreases recognition of latently infected cells by down regulation of MHC class I molecules, particularly in cells derived from Burkitt's lymphoma (Hislop et al., 2007).

MHC class II recognition is dampened by HHV-8 infection. Sabbah et al. reported that LCL, with an intact MHC class II processing pathway, could present LANA peptides to LANA-specific CD4^+^ T cell clones, whereas PEL cells were not recognized in an IFN-γ ELIspot (Sabbah et al., 2012). PEL express vIRF3, a known inhibitor of the MHC class II master regulator CIITA (class II transactivator) (Schmidt et al., 2011). When CIITA function was restored in PEL, CD4^+^ T cell clone recognition was also restored (Sabbah et al., 2012), supporting a role for HHV-8 in the reduction of MHC class II expression. Interestingly, IFN-γ inducible expression of CIITA results in MHC class II expression on endothelial cells, and is impaired after HHV-8 infection through induction of suppressor of cytokine signaling 3 (SOCS3) (Butler et al., 2012). This results in inhibition of the early events in the IFN-γ signaling pathway.

In sum, various HHV-8 proteins appear to play a significant role in the disruption of antigen processing and presentation. However, further data are needed to understand the extent of viral protein function in immunopathogenesis of HHV-8 infection in APC.

### T cell responses to HHV-8: relation to HHV-8 disease progression

Although immunity to HHV-8 is far less well-defined than that to EBV, T cell immunity to HHV-8 likely plays a similar, critical role in viral control. First, there is an increase in CD4^+^ and CD8^+^ expanded T cells in patients with classic KS that share a TCR-β variable subunit bias (Galleu et al., 2012), a phenomenon observed in response to chronic viral infections (Trautmann et al., 2005; Wynn et al., 2010). Second, CD8^+^ T cell immunity to HHV-8 proteins is present in HHV-8 seropositive, healthy individuals. CD8^+^ T cells specific for 5 HHV-8 lytic cycle proteins are present in blood in the first few months of primary HHV-8 infection of normal adults (Wang et al., 2001). This primary CTL and IFN-γ response to HHV-8 peaks within 2 years of infection, and wanes thereafter to low but detectable levels. Furthermore, KS does not commonly occur in HIV-1 infected individuals with high CD4^+^ T cell counts (Strickler et al., 1999).

To date, however, there is little direct evidence for a role of T cell immunity in HHV-8 infection and control of KS (Hislop and Sabbah, 2008). Lower CD8^+^ T cell responses have been found in persons with KS compared to asymptomatic persons (Guihot et al., 2006; Lambert et al., 2006). However, very modest increases in CD8 T cell responses to HHV-8 immunodominant peptides are found in persons on ART (Wilkinson et al., 2002; Bourboulia et al., 2004). While progressive increases in HHV-8 load precede development of disease in HIV-1-infected persons (Campbell et al., 2000; Laney et al., 2007), evidence is lacking for a direct association between control of HHV-8 load and HHV-8-specific, T cell immunity (Guihot et al., 2006). Nevertheless, an increased incidence of KS in organ transplant recipients and HIV-1-infected persons (Dedicoat and Newton, 2003) suggest a role for T cell immunity in prevention of KS, similar to T cell immunity in EBV-related cancers (Gottschalk et al., 2005). Reduction of immunosuppressive regimens can result in spontaneous resolution of KS in organ transplant recipients (Firoozan et al., 2005). Similarly, the incidence of KS has declined after suppression of HIV-1 by ART (Rabkin, 2001), where T cell numbers and function are partially restored (Rinaldo et al., 2000; Letvin and Walker, 2003; Benito et al., 2004). There are also shorter incubation periods for development of KS after HHV-8 infection in HIV-1-infected men compared to men infected with HHV-8 prior to HIV-1 infection (Gao et al., 1996; Jacobson et al., 2000). Primary infection with HHV-8 in immunosuppressed persons has a more severe outcome than reactivated HHV-8 infection. Finally, HHV-8 expresses many proteins that have immunomodulatory functions that could down-regulate T cell immunity (Areste and Blackbourn, 2009).

The emerging biology of KS and HHV-8 infection presents intriguing factors that interrelate HHV-8-specific T cell immunity to control of the cancer. HHV-8 is found as a latent infection in most of the spindle cells in the KS lesion (Moore and Chang, 1995; Foreman et al., 1997; Dupin et al., 1999; Boshoff and Chang, 2001). Since replication of herpesviruses in susceptible cells results in cell death, latency must be established either very soon after infection or possibly following an abortive (non-productive) infection. A small percentage of endothelial and KS spindle cells express a complete replication library of HHV-8 proteins early in the disease, whereas the majority of the transformed cells ultimately express only HHV-8 latency proteins. Circulating B cells and monocytes can be positive for HHV-8 DNA (Ambroziak et al., 1995; Blasig et al., 1997), and HHV-8-infected macrophages are present in KS tissues (Blasig et al., 1997). Th1 cytokines have been implicated in reactivation and persistence of HHV-8 in B cells and monocytes from KS patients (Sirianni et al., 1998). T cell infiltrates are common in KS tissues (Blasig et al., 1997; Fiorelli et al., 1998). CD8^+^ T cells in KS tissues produce IFN-γ and express HLA DR (Fiorelli et al., 1998; Sirianni et al., 1998), suggesting that tumor-infiltrating lymphocytes are responding to HHV-8 antigens.

Comprehensive longitudinal studies are needed to accurately assess the role of anti-HHV-8 T cell immunity in development of KS. T cell responses to HHV-8 could be directed at different lytic and latency proteins at different stages of infection and disease, similar to EBV (Gottschalk et al., 2005; Hislop and Sabbah, 2008). By comparison, the immediate early regulatory protein BMLF1 and other early and late lytic cycle proteins are targets for CD8 CTL during primary and latent EBV infection (Bogedain et al., 1995; Steven et al., 1997; Hislop et al., 2007).

### T cell responses to HHV-8: relation to T cell responses to EBV

We propose that T cell immunity in EBV infection provides lessons for what is likely occurring in HHV-8 infection. During mononucleosis caused by a primary infection with EBV, T cells specific for both lytic and latency EBV proteins are present, but responses to lytic epitopes tend to be stronger (Long et al., 2011). In healthy EBV seropositive individuals, CD8^+^ T cell responses are also found to be greater for lytic epitopes, with up to 3% of cells specific for a single lytic epitope and up to 0.5% for a single latency epitope (Hislop and Sabbah, 2008). Anti-EBV CTL responses shift during latent infection to EBV nuclear antigens EBNA3 and LMP2, while still retaining specificity for some lytic cycle proteins (Hislop et al., 2002). The hierarchy of CTL responses to immunodominant epitopes of EBV is related to a lower expression of latency proteins in infected cells (Pudney et al., 2005). Although HHV-8 does not have genes homologous to EBNA and LMP, HHV-8 latency-associated nuclear antigen (LANA or ORF73), kaposin (T0.7 or ORF K12), and K1 are putative latency and transforming proteins that are targets for CTL (Osman et al., 1999; Brander et al., 2001; Lepone et al., 2010).

Host selection of CD8^+^ T cell epitopes within HHV-8 proteins could be based in part on the relative expression of viral proteins by the MHC class I endogenous pathway, comparable to EBV (Levitsky et al., 1996). However, evidence from the anti-EBV CTL field indicates that CTL reactivity to this gamma herpesvirus varies as to the HLA haplotype, with different MHC class I haplotypes exhibiting different CTL reactivity to the same EBV proteins (Hislop et al., 2007). Perhaps HHV-8 has mechanisms similar to the Gly-Ala (Popescu et al., 2003) repeat domain in EBNA1 that inhibits proteosome processing of viral proteins through the MHC class I pathway (Levitskaya et al., 1995; Hislop et al., 2007), thereby inhibiting generation of EBNA1-specific T cells. In fact, LANA1 can inhibit protein processing *in cis* (Kwun et al., 2007; Zaldumbide et al., 2007). Bioinformatic analysis of HHV-8 sequences supports that latency proteins are likely to be poorer targets for CTL than immediate early or lytic proteins (Vider-Shalit et al., 2007). However, it is not yet clear if the *in cis* function of LANA1 is directly involved in down-regulation of CTL lysis of HHV-8 infected cells, including how it compares to other putative, *in trans* inhibitors of CTL function such as K3 and K5. Moreover, the EBNA1-CTL inhibition concept has undergone major revision. First, the GAr domains of EBNA1 can inhibit mRNA translation, which may be more critical to lack of CTL recognition than inhibition of proteosomal processing (Yin et al., 2003). Second, EBNA1 infected cells express EBNA1 peptides that can be recognized by CTL when assessed in more sensitive assays (Lee et al., 2004). This indicates that the effects of LANA1 on pathways related to CTL function that use chimeric constructs, indicator cell lines, etc., need to be characterized in a natural context using CD8^+^ CTL and natural targets that are specific for LANA1.

Similar to EBV, CD8^+^ T cell responses to HHV-8 tend to be directed toward lytic antigens (Robey et al., 2009). While there are much fewer CD8^+^ T cell epitopes known for HHV-8 than EBV, the majority of these epitopes are within the early and late lytic proteins (Robey et al., 2010, 2011). With regard to polyfunctionality, one study found that for both EBV and HHV-8, T cells specific for latency antigens were more polyfunctional than those specific for lytic antigens (Bihl et al., 2007b). The phenotype of these cells was also found to be different, with a greater proportion of effector memory T cells specific for latency antigens than lytic antigens for both EBV and HHV-8. In both EBV and HHV-8-associated malignancies, latency proteins are predominantly expressed, so it is thought that responses to latency proteins could be important in controlling these diseases (Hislop and Sabbah, 2008; Taylor and Blackbourn, 2011). Evidence suggests that there are higher levels of CD8^+^ CTL specific for EBV and cytomegalovirus (CMV) than HHV-8 in the blood of seropositive individuals (Wang et al., 2002; Guihot et al., 2006). Higher T cell responses to EBV and CMV antigens could be related to their greater viral load in persistently infected persons, with more turnover of viral antigen from latent, persistent reservoirs that maintains a greater level of memory CTL precursors.

Antigen-specific CD8^+^ T cells occupy a lineage of naïve and memory compartments that are involved in the expansion, effector, and contraction phases of CD8^+^ memory T cells (Halwani et al., 2006). Central memory and effector memory T cells are contrasted based on expression of surface molecules related to migration and differentiation. Patients with MCD have more CD8^+^CD45RA^−^CCR7^−^CD27^−^ IFN-γ^+^ cells (a late memory T cell phenotype) and fewer CD8^+^CD45RA^−^CCR7^−^CD27^+^ cells (early and intermediate T cell phenotype) than normal, HHV-8 seropositive controls. This phenotypic shift is not found for EBV-specific CD8^+^ T cells. Interestingly, HHV-8 viral loads are negatively correlated with early and intermediate effector memory cells. The more differentiated T cell phenotype is associated with disease, rather than a loss of HHV-8-specific CD8 T cells or polyfunctional activity, as the HHV-8-specific T cells are similar in function (secretion of IFN-γ, TNF-α, MIP1-β, and/or CD107a) in infected patients and healthy controls (Guihot et al., 2008).

In healthy, HHV-8 seropositive individuals controlling infection, there are both monofunctional and polyfunctional CD8^+^ T cells present that are specific for HHV-8 proteins (Lepone et al., 2010). This could have important implications in the immunopathogenesis of HHV-8 and for HHV-8-related disease development. In fact, patients who control KS have more polyfunctional CD8^+^ T cells producing IFN-γ and TNF-α, while patients with progressive KS have weaker and less polyfunctional HHV-8-specific CD8^+^ T cells (Bihl et al., 2009). IFN-γ-producing CTL specific for some HHV-8 lytic and latency proteins also express CD107 and TNF-α (Bihl et al., 2007b). This is similar to polyfunctional CTL that produce multiple cytokines such as IFN-γ, IL-2, and MIP-1β that are associated with enhanced control of HIV-1 infection (Betts et al., 2006; Makedonas and Betts, 2006; Streeck et al., 2008). Possibly relevant to these HHV-8-related factors is that CD8^+^ CTL specific for EBV lytic and latency proteins differ in phenotype, including expression of programmed death-1 (PD-1) (Hislop et al., 2007). PD-1 expression could act as a negative regulator of HHV-8-specific CD8^+^ T cells during disease progression.

While both monofunctional and polyfunctional antiviral CD8^+^ T cells are present in healthy HHV-8 seropositive individuals, a week-long DC-enhanced system is required to reveal these responses to HHV-8 proteins (Wang et al., 2002). Overall, the immune response to HHV-8 is relatively non-robust compared to T cell reactivity to other herpesviruses such as EBV (Bihl et al., 2007b; Lepone et al., 2010). This suggests that the number and/or functional capacity of circulating anti-HHV-8 T cells are relatively low. However, using direct staining with multimers of MHC class I molecules bound to nominal antigens, we have found that there is an average of 0.05–0.10% circulating, CD8^+^ T cells specific for single, immunodominant MHC class I epitopes of HHV-8 in healthy, HHV-8 seropositive individuals (Lepone et al., 2010).

It is possible that HHV-8-specific T cells are functionally down-regulated by T regulatory cells (Treg). Treg are operative in peripheral tolerance and beneficial in preventing autoimmunity and tissue damage, through such activities as inhibitory cytokine secretion and suppression of DC function (Vignali et al., 2008). However, Treg can also inhibit immunity needed to resolve infections. While little is currently known about Treg during HHV-8 infection and disease development, these cells have been found to be important during other viral infections, including EBV (Li et al., 2009) and HIV-1 (Macatangay and Rinaldo, 2010). During primary EBV infection, patients with mononucleosis have less Treg than healthy seropositive individuals (Wingate et al., 2009). In patients with Hodgkin's lymphoma, Treg accumulated at tumor sites and those patients with higher Treg ratios had shorter disease-free survival (Marshall et al., 2004; Schreck et al., 2009). Additionally in these patients, several EBV epitopes stimulate Treg, and the increases in Treg numbers are associated with decreased EBV-specific CD8^+^ T cell IFN-γ production (Marshall et al., 2007). During HIV-1 infection, HIV-1-specific CD8^+^ T cell responses and cytolytic activity are repressed by Treg (Kinter et al., 2007). Immunotherapy with HIV-1 peptide-loaded MDDC enhances Treg function, resulting in inhibition of HIV-1 specific, CD8^+^ T cell polyfunctionality (Macatangay et al., 2010). Importantly, this antiviral function can be restored *in vitro* by depleting Treg. In patients with nasopharyngeal carcinoma, large numbers of Treg are found both at tumor sites and in circulation (Lau et al., 2007; Li et al., 2007a). As these cells could also be important in HHV-8-related disease development such as KS, studies are needed to determine their exact role.

## Summary

Amodio et al. recently showed that the presence of virus-specific T cells against LANA in classic KS patients was associated with persistent KS, while K8.1-specific T cells were inversely correlated with KS occurrence (Amodio et al., 2011). Robey et al. reported a novel late-lytic glycoprotein ORF28-P29 epitope that was recognized in 7% of HIV^+^/HHV-8^+^ individuals (Robey et al., 2011), compared to an immunodominant HLA A^*^0201 late-lytic glycoprotein K8.1 (Bourboulia et al., 2004) recognized in 71% by IFN-γ ELISPOT. Using pentamers, ORF28-P29-specific CD8^+^ T cells were determined to have an effector memory phenotype. Given these results, it appears that T cell responses, in both quality and magnitude, are essential for control of HHV-8 infection. Although very intriguing, these studies regarding the presence, frequency, and phenotype of HHV-8-specific T cells in HHV-8 infected, and HHV-8/HIV-1 coinfected persons are primarily cross sectional. This lacks the power of more revealing, multi-dimensional longitudinal analysis of anti-HHV-8 T cell responses. For example, it was recently demonstrated that a decline in the quality of HIV-1-specific CD4^+^ and CD8^+^ T cells in HIV-1 infection, including functional cytokine production, and a shift toward a memory cell phenotype, occurred over a 7 year period (Dembek et al., 2012). This evidence supports the need for longitudinal studies focusing on the reactivity of CD8^+^ T cells to HHV-8 lytic and latency cycle proteins. We speculate that CD8^+^ T cell responses in those individuals that control infection will vary from those who develop KS. Such studies will provide insight to the role of these effector responses and how they contribute to the prevention of KS.

## Conclusion

To succeed, a pathogen must be able to evade immune surveillance. In this review, we have described the effect of HHV-8 infection on cells of the immune system, with particular emphasis on professional APC and the subsequent effect on T cell responses. Recognition that DC-SIGN expressed on DC, macrophages and B cells acts as a major receptor for HHV-8 has enhanced our ability to assess the effect of HHV-8 infection of these primary cells. This has revealed two distinct replication patterns of HHV-8 in APC, i.e., non-productive and productive, which could have direct consequences on viral pathogenesis. Furthermore, this should enable studies of virus gene transcription cascade in cells capable of supporting productive infection that are natural targets of HHV-8. Studies have also begun to elucidate the effect of HHV-8 infection on DC and B cell functions, as measured by cytokine and chemokine production and impairment of antigen presentation. The direct effect of HHV-8 infection of professional APC and its indirect effect on T cell control of infection are being tied together in a more revealing fashion to define the magnitude and breadth of T cell responses to HHV-8 antigens. T cell responses to HHV-8 antigens are not very robust as compared to EBV and CMV. The dampened immune response observed upon HHV-8 infection could be related to Treg activity. Although evident in HHV-8 infection, it is not clear whether polyfunctional T cells are required to control progression of associated diseases. Given that the most common route of HHV-8 transmission is through saliva, and that KS lesions predominate in the skin and mucosa, APC from mucosal sites are likely to be critical in controlling HHV-8 transmission and pathogenesis. Understanding how these events are influencing the ability of APC to induce an effective immune response is essential in the development of therapeutic and preventative vaccine strategies.

### Conflict of interest statement

The authors declare that the research was conducted in the absence of any commercial or financial relationships that could be construed as a potential conflict of interest.
